# Dielectrophoretically Assembled SWCNTs Networks on SU-8 Substrate for PEG/SWCNTs Composite Gas Sensor

**DOI:** 10.3390/polym16010074

**Published:** 2023-12-26

**Authors:** Jin-Chern Chiou, Chin-Cheng Wu, Tse-Mei Lin, Yu-Chieh Huang

**Affiliations:** 1 Department of Electronics and Electrical Engineering, National Yang Ming Chiao Tung University, 1001 University Road, Hsinchu City 30010, Taiwan; 2 Institute of Electrical and Control Engineering, National Yang Ming Chiao Tung University, 1001 University Road, Hsinchu City 30010, Taiwan

**Keywords:** single-walled carbon nanotubes, polyethylene glycol, SU-8, dielectrophoresis, gas sensor

## Abstract

This study proposed a SU-8 based gas sensor, integrated with heater and sensing electrodes, to develop a multi-channel gas sensor with PEG/SWCNTs composite films. The impedance of single-walled carbon nanotubes (SWCNTs) on each sensing electrode was well controlled via dielectrophoresis technology. To investigate dielectrophoretic mobility characteristics, the concentric circular sensing electrode has three different spacing between the inner and outer electrodes, including 10 μm, 15 μm, and 20 μm. The electrodes were applied with a 5 MHz AC source with a voltage ranging from 1 Vpp to 5 Vpp. Polyethylene glycol (PEG) was deposited on the gas sensor via drop casting. The fabricated gas sensor was operated at different working temperatures, including 25 °C, 40 °C, 50 °C and 60 °C, to examine the sensing response. The response results revealed that the PEG/SWCNTs composites gas sensor with 60 °C working temperature exhibited the ability to detect 80 ppm ethanol vapor.

## 1. Introduction

Both conductive polymer sensors and carbon nanotubes (CNTs) sensors have been investigated extensively in the past few years for the application of vapor sensing, due to their unique electrical properties and diverse responses to different gases. The polymer matrix materials with electrically conductive fillers exhibit diverse stimuli-responsive behavior and conductivity responses when exerting external environmental stress, such as vapor, temperature, humidity and pressure. Due to their high sensitivity, cost-effectiveness, low power consumption, and manufacturability, they have been widely used for sensor applications in environmental detection and monitoring [1,2,3]. Polymer/carbon nanotube composite sensors integrated with their advantages use a physical adsorption mechanism to provide the ability to sense gas molecules. This type of sensor array made of different polymer materials constructs a unique response pattern for the vapors which has the potential to distinguish different gases [4,5]. When odor with ambient temperature variation is introduced into polymer/CNTs composite sensors, it induces swelling and percolation and then the charge transportation exhibits chemiresistive properties. The ambient temperature effect on the polymer matrix is attributed to the tiny changes in the cell walls of the host polymer, which affect the conductive network. However, the variation in ambient temperature is a critical problem when it comes to reducing the sensitivity of the sensor [6,7]. In our previous studies [8,9], we examined the effect of the operating temperature dependence on electrical characterization and sensor response and then addressed the ambient temperature interference issue by using a thermostat with a moderate operating temperature which provided uniform thermal distribution.

Drop casting polymer/CNTs composite on a substrate with electrodes is one of the cost-effective methods used to fabricate the polymer-based gas sensor. However, the carbon nanotube agglomeration effect led to uneven dispersion and unstable concentration of CNT solution, resulting in unstable electron conductivity of polymer/carbon nanotube composite gas sensors and obtaining an unstable response, longer desorption times, and significant baseline drift issues when introduced gas. In addition, the inconsistent impedance among the individual sensors in the gas sensor array will indeed complicate the design of the sensing interface circuit [10,11].

Dielectrophoresis (DEP) is one of the effective methods used to reproducibly control the linear density of the CNTs between electrodes. DEP is a translational motion caused by the polarization effect in a non-uniform electric field. The CNTs alignment using DEP is based on three mechanisms of the CNT dielectric polarization, including (1) the electronic polarization mechanism, which appears when the electron cloud is relatively far from its charge center compared to the nuclear charge; (2) the atomic or ionic polarization, which occurs when the ions or atoms of different charges move in opposite directions; (3) the orientational polarization takes place when the molecules with permanent dipole moments align in a polar dielectric [12]. There have been many studies on the deposition and arrangement of carbon nanotubes [13,14,15]. The external electric field generates dielectrophoretic forces and torques, inducing dipole moment forces on nanotubes suspended in a dielectric medium. Several parameters influenced the dielectrophoretic assembly of carbon nanotubes, including the geometry of the electrode, the applied amplitude and frequency property of AC signal, and the conductivity and material properties of the carbon nanotubes and the dielectric medium.

In this paper, we present the flexible gas sensor array with the high-density alignment of single-walled carbon nanotubes (SWCNTs) via dielectrophoresis. The proposed gas sensing array was fabricated on the SU-8 substrate and composed of the selected PEG/SWCNTs composite sensing films, concentric sensing electrodes, and heater. This study manipulated the resistance values of SWCNTs in the sensor by adjusting the voltage amplitude and frequency parameters of the electric field at different electrode spacing.

## 2. Materials and Methods

### 2.1. CNTs/Polymers Composite Gas Sensor Architecture and Fabrication

**Figure 1:**
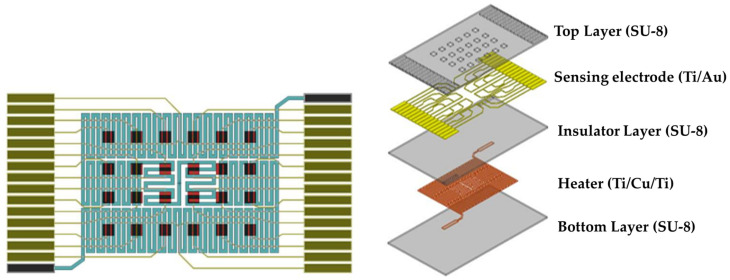
Schematic diagram of PEG/SWCNTs composite gas sensor (**left**) and explosion drawing (**right**).

The SU-8-based gas sensor array comprised 24 gas sensors arranged in a 6 × 4 matrix pattern. The structure of the PEG/SWCNTs composite gas sensor in this study was composed of five components: a substrate (SU-8), a heater (Ti/Cu/Ti), a sensing electrode (Ti/Au), and a PEG/SWCNTs sensing film, as illustrated in Figure 1.

**Figure 2:**
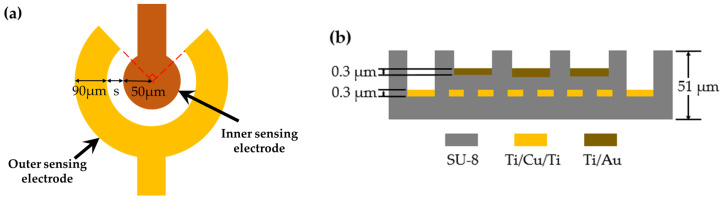
(**a**) Configuration of the single circular sensing electrode. (**b**) the cross-sectional schematic structure.

The configuration of the circular sensing electrode and the cross-sectional view of the gas sensor are shown in Figure 2. The single sensing circular electrode consists of a circular inner sensing electrode with a diameter of 50 μm and an annular outer sensing electrode with a 90° notch and a width of 90 μm. There are four different configurations of electrode spacing (denote: s): 10 μm, 15 μm, 18 μm and 20 μm. The sensing electrodes are composed of a stack of Ti/Au with a thickness of 0.3 μm. A PEG/SWCNTs composite film is filled in specific areas on top of the sensing electrodes. The heater made of Ti/Cu/Ti has a 0.3 μm thickness, 300 μm line width, and a geometry corresponding to 30.4 mm × 15.8 mm.

**Figure 3:**
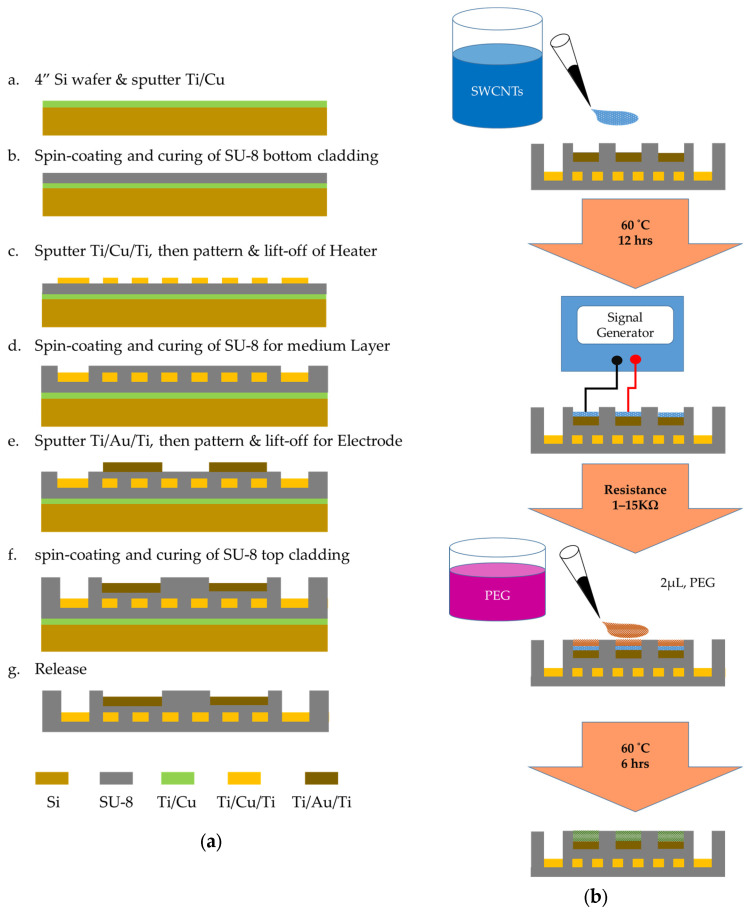
Schematic illustration of fabricating process. (**a**) Gas sensor based on SU-8 that incorporates Ti/Cu/Ti micro heater and Ti/Au/Ti sensing electrodes. (**b**) Fabrication process of the PEG/SWCNTs sensing film.

The fabrication process of the SU-8 based gas sensor is shown in Figure 3, which includes the following steps:

(a) A 4″ silicon wafer used as the supporting plate of the flexible SU-8 film was cleaned via the RCA process.
(b) DC sputter deposition of adhesion layer (500 Å Ti) and sacrificial layer (3000 Å Cu) occurred.
(c) Spin coating and curing of 17 μm SU-8 bottom cladding was performed.
(d) Spin coating with positive photoresist AZ4620 (AZ Electronic Materials, Bridgewater, NJ, USA) was performed, followed by heating at 90 °C for 2 min, then the 1st mask and photolithography process was conducted. Then, we used a DC vacuum sputtering system to deposit 500 Å of titanium (Ti), 2000 Å of copper (Cu), and 500 Å of titanium (Ti). After deposition, we used a lift-off process for the pattern of the heater.
(e) After spin coating with negative photoresist SU-8 3025 with 17 μm, we performed the 2nd mask and photolithography process. Spin-coating with a thickness of 5 μm of positive photoresist was performed, followed by heating to 90 °C for 2 min, and the silicon wafer was immersed in the developer (Propylene glycol methyl ether acetate, PGMEA) for 1 min.
(f) After spin coating 5 μm positive photoresist AZ4620, we performed the 3rd mask and photolithography process. We used a DC vacuum sputtering system to deposit 500 Å of titanium (Ti), 2000 Å of gold (Au), and 500 Å of titanium (Ti). We used a lift-off process to remove excess metal and generate the designed electrode, wiring and pad.
(g) After spin coating 17 μm negative photoresist SU-8 3025 and heating to 95 °C for 30 min, we immersed the silicon wafer in the developer (PGMEA) for 1 min, then heated it to 120 °C for 10 min. We performed the 4th mask and photolithography process process to complete the electrode pad opening. We used a Reactive Ion Etching (RIE) system for anisotropic dry etching of the top layer of titanium, defining the region where the electrode needed to be electrically isolated on the pad.
(h) We immersed the component in copper etchant to release the sensor from the slicon wafer.
(i) After the SU-8 based structure was fabricated, the PEG/SWCNTs bilayer sensing films were prepared via dielectrophoresis and drop casting method.

To assess the sensing properties of PEG/SWCNTs composite film, we fabricated sensing film via drop casting and dielectrophoresis. At first, the 4 μL of SWCNTs (100 ppm) was dropped on the concentric circular electrode using a micropipette. The SWCNTs were purchased from Golden Innovation Business, New Taipei City, Taiwan, and were fabricated using the chemical vapor deposition method and have outer diameters ranging from 1 to 1.5 nm and lengths of 1 to 5 μm.

**Figure 4:**
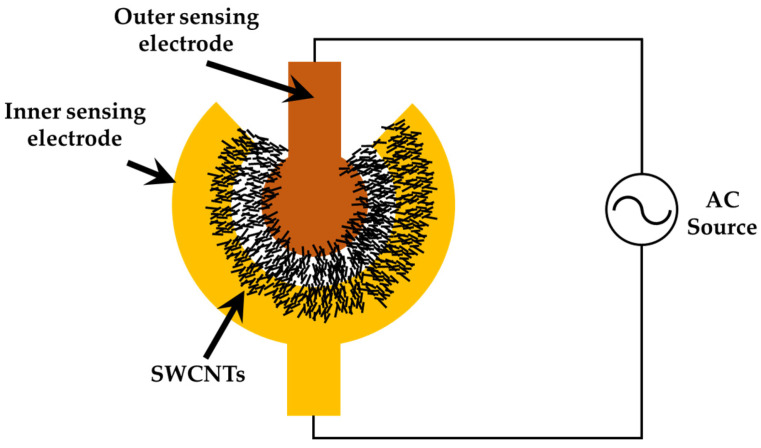
Experimental setup for the dielectrophoresis of SWCNTSs. The AC voltages were connected to sensing electrode pads and then applied to SWCNTs solvent during the subsequent DEP process.

In the dielectrophoresis process, the electrode is connected to a signal generator (AFG-3102, Tektronix, Beaverton, OR, USA), as illustrated in Figure 4. A sinusoidal voltage with a peak-to-peak voltage from 1 Vpp to 5 Vpp operated at 5 MHz was applied to the electrodes until the resistance was satisfied in the designated range between 1 kΩ and 15 kΩ. After that, the sensor was baked at 60 °C for 12 h.

According to the linear solvation energy relationship (LSER) theory and physisorption mechanism [16,17], the polyethylene glycol (81260, SIGMA-ALDRICH, St. Louis, MO, USA) was selected to detect various target gases. PEG (0.2 g) was dissolved in 100 mL tetrahydrofuran (THF). The PEG solution was prepared via sonication for 6 h in an ultrasonic bath at room temperature for homogenization.

**Figure 5:**
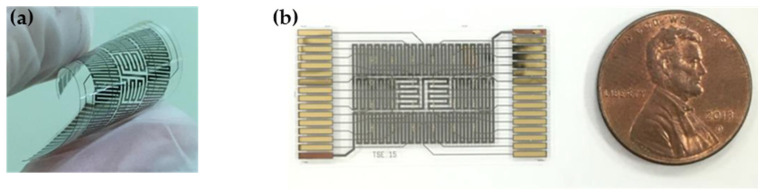
Schemes of SU-8 based gas sensor; (**a**) configuration of the flexible gas sensor array; (**b**) the packaged gas sensor for gas experiment.

The 2 μL PEG solution was dropped casting on the sensing electrode and baked at 60 °C for 6 h to form a two-layer structure of single-walled carbon nanotube/polymer composite sensing film. The proposed SU-8 based gas sensor exhibited excellent flexibility [18]. Figure 5 shows the sensor was packaged via the PCB gas response experiment.

### 2.2. Experimental Scheme for Gas Sensor Evaluation

**Figure 6:**
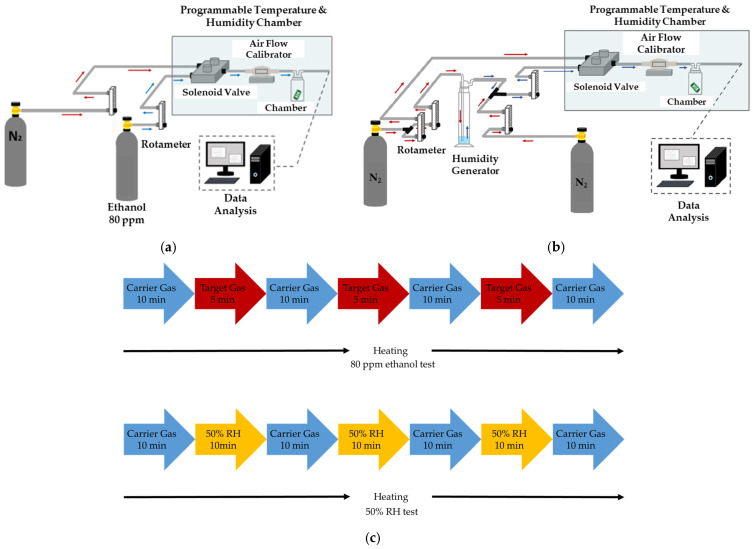
Experimental setup of the gas sensing system. (**a**) Experimental setup of the 80 ppm ethanol test; (**b**) experimental setup of the humidity test; (**c**) gas testing procedure.

The experimental setup of the humidity test is illustrated in Figure 6a. The target gas and carrier gas were both controlled by mass flow controllers with a flow rate of 600 mL/min. High-purity nitrogen (N_2_, ≥99.99%) gas was selected as the carrier gas to desorb the target gas. The PEG/SWCNTs composite gas sensor and humidity and temperature sensors were placed in the chamber with 60 mL capacity.

To understand the humidity influence on the sensing film, the humdity generator bubbled nitrogen through distilled water to generate a humidity of 50% RH to examine the impact of humidity on the film. The experimental setup of the humidity test is illustrated in Figure 6b.

The gas response experiment consists of several steps in each cycle. Firstly, the heater was operated at a specific temperature, and then the carrier gas was introduced for 10 min to establish a reference resistance baseline. Establishing a baseline allows for the identification of any background signals or interference, providing a reference point for comparing subsequent measurements with gas exposure. Subsequently, the target gas was introduced for 5 min. This step provided information on how the sensor interacts with the target gas, leading to the detection and measurement of the gas concentration. The polymer membrane adsorbs and expands due to gas molecules. Afterward, the carrier gas was introduced for 10 min to achieve desorption from the polymer film. Desorption served the sensor for subsequent measurements by clearing the sensor surface from any remnants of the previously adsorbed gas, ensuring a clean slate for the next cycle. Repetition is essential for obtaining reliable and statistically significant results. It also helped us understand the sensor’s performance characteristics under continuous or cyclic exposure to the target gas. The aforementioned steps were repeated at least two times to examine the repeatability and desorption performance.

The aforementioned cycle was repeated three times at a specific temperature to understand the relation between operating temperature and gas-sensing properties. In this study, the operating temperatures were tested at 25 °C, 40 °C, 50 °C, and 60 °C. Figure 6c shows the gas testing procedure.

## 3. Results and Discussion

### 3.1. Dielectrophoresis Characterization

SWCNTs generated an induced dipole moment due to the external electric field provided by the alternating current power source. The electrons and protons in the SWCNTSs were separated and moved toward their balanced positions. The polarizability of the SWCNTs was determined by the geometry of SWCNTs and the difference in the complex permittivities of the suspending medium. The dielectrophoretic force F existing between the dipoles and the electric field can be expressed as [19]:

(1) F=πr2l6εmReKf∇E2

where *l* and r are the geometry factors which related to the length and the molecular radius of the single CNT, $\varepsilon_{m}$ is the permittivity of the suspending medium and E is the electric field. The factor $K_{f}$ depends on the complex permittivities of both the particle and the medium. Re{$K_{f}$} is referred to as the Claussius–Mossotti factor. The “positive” DEP force occurs when the real part of the particle’s complex permittivity is greater than the fluid medium, and is directed toward the point where the field strength is highest. In contrast, “negative” DEP forces occur when the real part of the particle’s complex permittivity is smaller than the fluid medium and is forced away from regions of high field strength. The real part of the Clausius–Mossotti factor determines the particle will experience a positive or negative DEP force. According to the dielectrophoretic force, the SWCNTs are able to align along the directions of the electric field lines in the solution.

**Figure 7:**
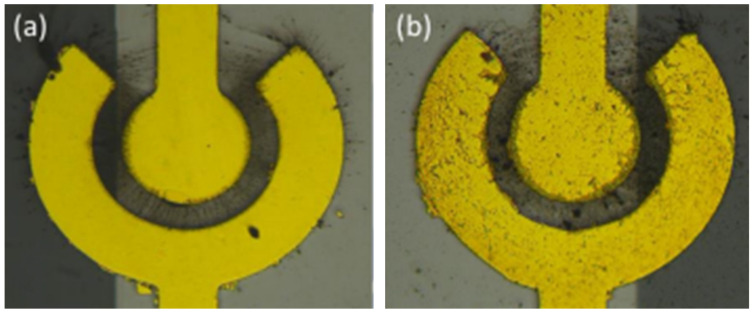
Schematic of DEP process (**a**) the AC source electric field was applied on the electrode to align the SWCNTs; (**b**) the electrode deposited SWCNTs after the DEP process.

There are some parameters related to dielectrophresis, including the intensity of the AC voltage and its sinusoidal frequency, the concentration and properties of SWCNTS and the duration time of DEP. To investigate electrophoretic mobility characteristics, the concentric circular sensing electrode has three different spacings between the inner and outer electrodes: 10 μm, 15 μm, and 20 μm. The electrodes were applied to a 5 MHz AC source with a voltage ranging from 1 Vpp to 5 Vpp. When an external voltage generates a electric field at the sensing electrodes, it provides the necessary energy for the dielectrophoresis of SWCNTs. The SWCNTs were moved by dielectrophoretic force and aligned along the electric field line to bridge the inner and outer electrodes. Figure 7 illustrates the SWCNTs during the DEP process and after the process.

The gap between concentric electrodes influences the dispersion and electrical properties of SWCNTs. After dielectrophoresis, the resistance of the concentric circular sensing electrode is listed in Table 1.

**Figure 8:**
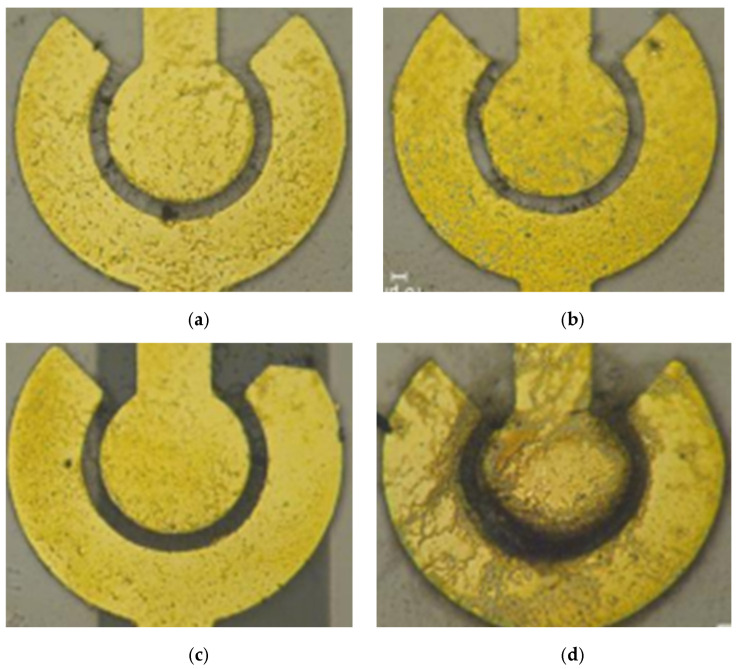
The distribution of SWCNTs on the electrode after applying the different peak-to-peak value of an AC to the electrode. (**a**) 2 Vpp; (**b**) 3 Vpp; (**c**) 4 Vpp; (**d**) 5 Vpp.

When there is a large electric field with high frequency or amplitude, SWCNTs have enough energy to move toward the electrode. However, the unexcepted electrothermal force on the medium could influence the conductivity and permittivity [20]. The resistance decreases as the voltage increases; the voltage is related to the electric field intensity, and higher voltage leads to a greater number of SWCNTs being bridged. Figure 8 shows the distribution of SWCNTs on the electrode after applying the different peak-to-peak value of an AC to the electrode.

**Figure 9:**
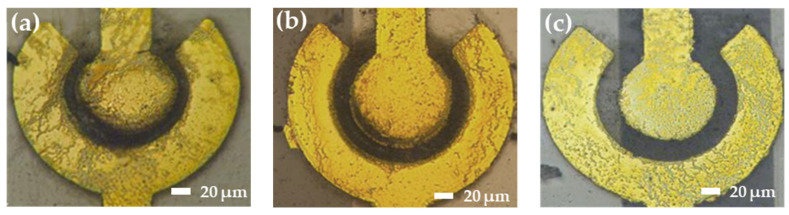
The distribution of SWCNTs in different gaps between the inner and outer electrodes at 5 Vpp with 5 MHz. (**a**) 10 μm, (**b**) 15 μm, and (**c**) 20 μm.

When the same external voltage is applied, the bridging capability decreases with an increasing gap distance. As the distance between the inner and outer electrodes increases, the weak electric field is unable to supply sufficient energy to move the SWCNTs. Figure 9 shows the distribution of SWCNTs in different spacings between the inner and outer electrodes at 5 Vpp with 5 MHz.

Based on the different gap sizes, appropriate voltages are selected to achieve the optimum impedance: 3 Vpp and 4 Vpp for a 10 μm gap, 4 Vpp and 5 Vpp for a 15 μm gap, and 5 Vpp for an 18 μm gap, maintaining the initial impedance in the range of 1 kΩ to 10 kΩ. As demonstrated by the images and impedance results, under the same voltage conditions, the bridging capability declined as the gap distance increased. The magnitude of the voltage was related to the electric field intensity, and a higher voltage resulted in a greater number of bridged nanotubes.

**Figure 10:**
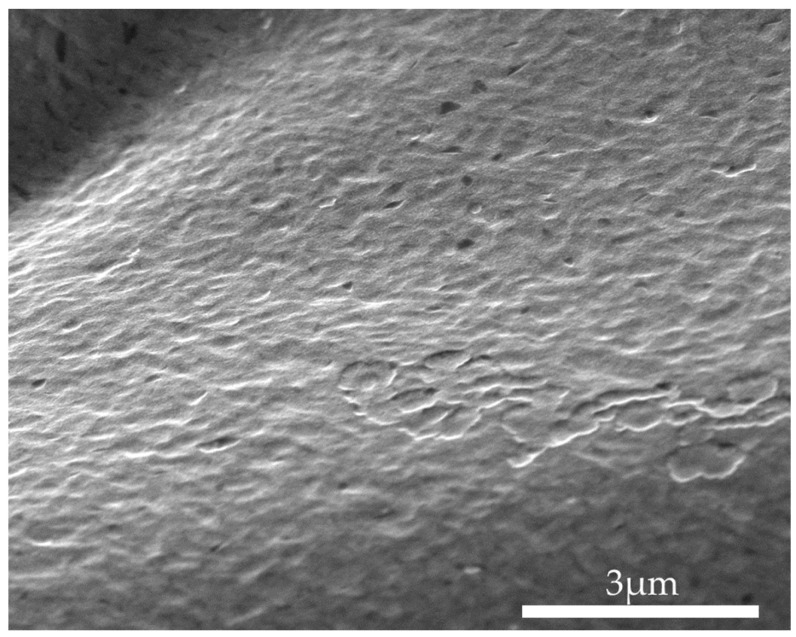
SEM morphologies of PEG/SWCNTs composite films.

The morphology of the PEG/SWCNT sensing film was examined using a Scanning Electron Microscope (NOVA NANO SEM 450, FEI Co. Hillsboro, OR, USA) at an accelerating voltage of 10 kV, and the morphology of the composite film was obtained, as shown in Figure 10.

### 3.2. Heater Performance

**Figure 11:**
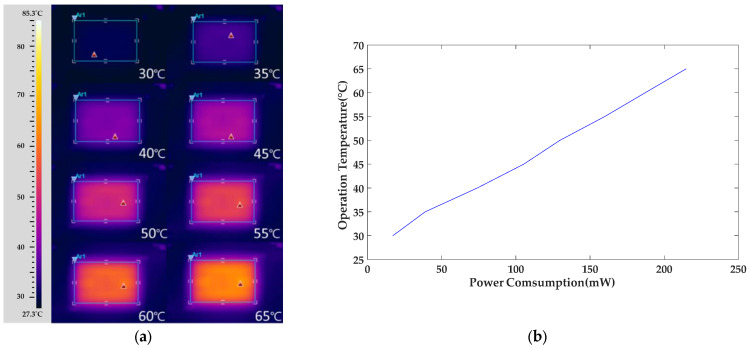
The heater synchronously provided different working temperature zones ranging from 30–65 °C with successive increments of 5 °C. (**a**) Infrared thermal image of thermal distribution. (**b**) Power consumption vs. operating temperature of the heater for the gas sensor.

The heating performance of the heater was verified using an infrared thermal imager (FLIR ThermoVision A320, TRANSCAT, New York, NY, USA) to ensure the uniform distribution of heat. The heater was supplied with different voltages from a power supply to heat the substrate. The operating temperature gradually increased from 30 °C to 65 °C, with thermal imaging conducted every 5 °C. The measurement results are shown in Figure 11.

Observations from the figure indicate that the heater exhibits good thermal distribution symmetry and uniformity when heated to 65 °C. Within the sensing range, the sensing electrodes are uniformly heated. The heater effectively controls the temperature in the sensing area, ensuring even heating of the sensing film. This uniform heating is crucial for gas detection, preventing uneven expansion of the sensing film due to uneven heating and thereby maintaining the stability and reproducibility of the gas sensor.

Additionally, to understand the actual electrical power consumption characteristics of the heater, the voltage supplied by the power source to heat the heater to the target temperature was recorded during the heating characteristic measurements. Combining this heater design, the arrayed PEG/SWCNTs gas sensor developed in this study can use the heating method to drive away moisture, reducing interference from environmental humidity. The heater can also operate at a fixed temperature, facilitating gas detection by the sensing film without being affected by changes in ambient temperature.

### 3.3. Response of Target Gas

Ethanol is a volatile, flammable, colorless liquid that can cause serious and permanent damage to the brain and other organs if consumed in excess. Therefore, it is important to develop a high-performance gas sensor that can detect low-concentration ethanol gas.

The fabricated gas sensor was tested via a gas-testing cyclic experiment to investigate the sensing response of 80 ppm ethanol with different operating temperatures [21]. The target gas (ethanol) was repeatedly introduced three times in the aforementioned process to observe the repeatability [22]. Since the carrier gas (nitrogen gas) introduced the gas sensor, the ambient and target gas were effectively removed, then a reference resistance baseline was obtained. Normalized resistance changes ($\frac{\Delta R}{R_{0}}\%$) of the sensor response were determined using $\frac{\Delta R}{R_{0}}\%=\left[\frac{R_{\max}-R_{0}}{R_{0}}\right]\times 100$, where *R*_0_ implies the reference baseline, which is the mean value of sensor resistance when the sensor is exposed to carrier gas in equilibrium, and *R*_max_ implies the maximum resistance when the sensor is exposed to ethanol.

**Figure 12:**
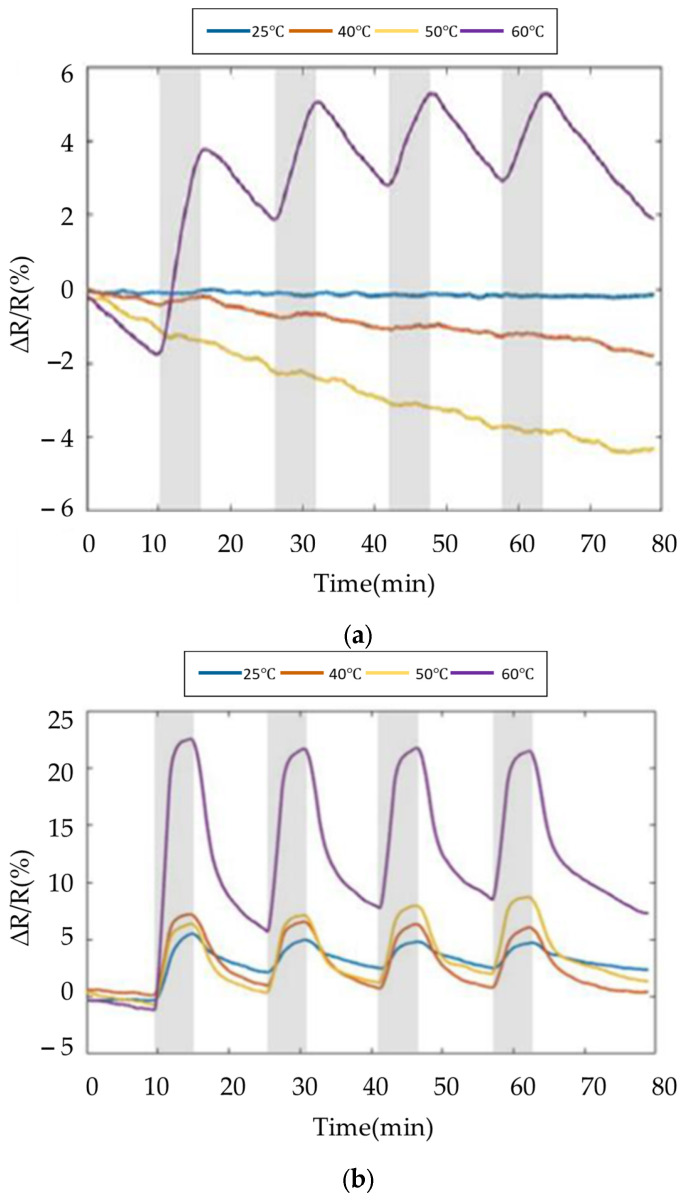
Response of the gas sensor array to 80 ppm ethanol at different temperature. (**a**) SWCNTs; (**b**) PEG/SWCNTs.

The results of SWCNTs and PEG/SWCNTs composite sensing film exposure to 80 ppm ethanol are shown in Figure 12. The sensitivity of the SWCNTs sensing film is about 2.3% to ethanol at 60 °C, but is not apparent at other temperatures. In Figure 12b, the sensitivity of PEG/SWCNT composite sensing film to ethanol is 2.9% at room temperature. As the operating temperature increases, the sensitivity also increases, reaching 15.6% at 60 °C. The ethanol response increases with the operating temperature. This implies that the favorable interaction between the PEG surface and the target gas molecules induces charge transfer in SWCNTs. The increased response may be attributed to the moderate operating temperature, which facilitates the thermal degradation of PEG side chains [23]. The dielectrophoresis assembly of SWCNTs exhibits improved swelling properties, enhancing the van der Waals interactions and vapor molecules on the surface of the PEG/SWCNTs gas sensor [24,25].

**Figure 13:**
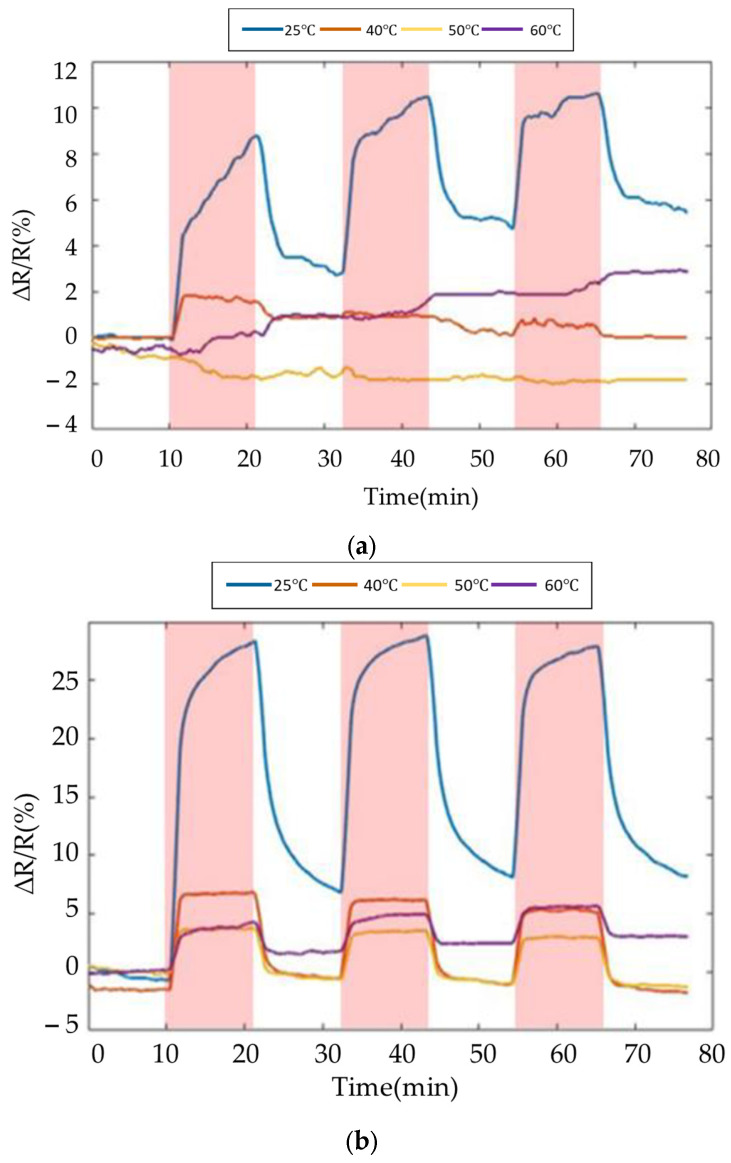
Response of the gas sensor array to 50% RH at different temperature. (**a**) SWCNTs; (**b**) PEG/SWCNTs.

The experiment results of the SWCNTs and PEG/SWCNTs composite sensing film at 50% relative humidity are shown in Figure 13. At 25 °C, SWCNTs showed sensitivity to humidity, but when the temperature increased, no obvious response of SWCNTs to humidity was observed. PEG/SWCNT showed higher sensitivity to humidity, around 20.1%. However, as the temperature increases, the sensitivity decreases, reaching about 3% at 60 °C, but there is no obvious signal drift.

Due to the adsorption of water molecules on the nanotube surface, surface functional groups such as –OH and –COOH are generated [26]. These water molecules move to the SWCNTs and recombine with their electrons and holes. This recombination leads to a reduction in the majority carriers in the SWCNTs, resulting in an increasing resistance.

As the temperature of the sensing film increases, the carrier concentration rises, leading to more carrier recombination and an increase in conductivity, causing the resistance to decrease, and exhibiting a negative temperature coefficient resistance characteristic [27]. Furthermore, the results indicate that the PEG/SWCNTs composite film sensing enhances the charge transfer mechanism, demonstrating improved sensitivity and desorption capability.

## 4. Conclusions

In summary, we developed the gas sensor based on SU-8 substrate, combined with Cu microheaters and Au sensing electrodes, to create a flexible multi-channel gas sensor. Single-walled carbon nanotubes (SWCNTs) were manipulated by AC electrophoresis to bridge resistive elements between Au electrodes to achieve impedance control. Subsequently, PEG were drop-casted on SWCNTs to form a composite sensing film. The substrate was heated to different temperatures to examine the ethanol gas response of PEG/SWCNTs composite polymer sensing films. Under 80 ppm ethanol vapor concentration and at a 60 °C working temperature, it was found that the sensitivity of the PEG/SWCNTs ethanol gas sensor was 15.6%.

## Figures and Tables

**Table 1:** DEP Voltage and the resistance of each electrode spacing.

DEP Voltage (Vpp)	Electrode Spacing			
	10 μm	15 μm	18 μm	20 μm
1 V	44.8 K	132.4 K	1700 K	2300 K
2 V	29.6 K	69.8 K	237.7 K	330 K
3 V	16.6 K	23.6 K	57.8 K	68.9 K
4 V	1.65 K	11.3 K	23.4 K	33.1 K
5 V	0.991 K	2.43 K	7.1 K	23.7 K

## Data Availability

The data presented in this study are available on request from the corresponding author.
